# How drain flies manage to almost never get washed away

**DOI:** 10.1038/s41598-020-73583-2

**Published:** 2020-10-20

**Authors:** Nathan B. Speirs, Gauri A. Mahadik, Sigurdur T. Thoroddsen

**Affiliations:** Division of Physical Science and Engineering, King Abdullah University of Science and Technology (KAUST), Thuwal, 23955-6900 Saudi Arabia

**Keywords:** Entomology, Biological physics, Fluid dynamics

## Abstract

Drain flies, Psychodidae spp. (Order Diptera, Family Psychodidae), commonly reside in our homes, annoying us in our bathrooms, kitchens, and laundry rooms. They like to stay near drains where they lay their eggs and feed on microorganisms and liquid carbohydrates found in the slime that builds up over time. Though they generally behave very sedately, they react quite quickly when threatened with water. A squirt from the sink induces them to fly away, seemingly unaffected, and flushing the toilet with flies inside does not necessarily whisk them down. We find that drain flies’ remarkable ability to evade such potentially lethal threats does not stem primarily from an evolved behavioral response, but rather from a unique hair covering with a hierarchical roughness. This covering, that has never been previously explored, imparts superhydrophobicity against large droplets and pools and antiwetting properties against micron-sized droplets and condensation. We examine how this hair covering equips them to take advantage of the relevant fluid dynamics and flee water threats in domestic and natural environments including: millimetric-sized droplets, mist, waves, and pools of water. Our findings elucidate drain flies’ astounding ability to cope with a wide range of water threats and almost never get washed down the drain.

## Introduction

Water provides amazing opportunities for life at the interface, but can also pose potentially lethal threats to insects. The danger stems from the physics at fluid interfaces. The small length scale of most insects (described by some characteristic length $\ell$) causes the force of surface tension ($\propto \ell$) to exceed the force the organism can exert by either its strength ($\propto \ell ^2$)^1^ or weight ($\propto \ell ^3$). This means that if an insect’s appendage gets stuck in water, it lacks the strength or weight to extricate itself. The ratio of these forces demonstrates the dominant influence of surface tension as $\ell$ becomes small.

To mitigate the risk of surface tension, many insects and plants rely on specialized surface features that decrease water’s ability to adhere to or wet them; i.e. they become more hydrophobic so they do not get stuck in water^2–5^. Appropriate surface chemistry increases hydrophobicity, but alone is limited to producing a maximum solid-liquid contact angle $\theta _o$ of $120^{\circ }$^6^, which quantifies hydrophobicity^7^. Augmenting chemistry with roughness can increase the apparent contact angle^8^ $\theta _a$, and superhydrophobicity ($\theta _a > 150^{\circ }$) occurs when air becomes entrapped in the valleys of roughness elements (Cassie wetting) with increasing air fraction raising $\theta _a$^9^. Many insects take advantage of this wetting physics and attain superhydrophobicity by both coating their bodies with oil or wax to optimize their surface chemistry^3,10,11^, and using hierarchical roughness structures to maximize the air fraction (minimize the solid-liquid contact) between the liquid and their bodies^4^. The roughness appears in many forms in insects and plants alike, including: nanopillars on drone fly^12^ and dragonfly wings^13^, micropapillae on lotus leaves^14^, micropapillae with nanofolds on rose petals^15^, needle shaped setae with nanogrooves on water strider^11,16^ and crane fly legs^17^, and arrays of hair with star-shaped cuticular projections on termite wings^18^. Insect surface chemistry and roughness helps them to stay dry, but their interactions with water brings other challenges as well.

Surface tension and superhydrophobicity also allow many insects and spiders to stand and move on the surface of ponds and other bodies of water by supplying them with most of the static and dynamic weight support required^19–22^. If insects move too quickly when they are in search of food or to evade predators^23,24^, the dynamic pressure they generate overcomes the interfacial pressure and they pierce the interface with potentially hazardous results^25,26^. To deal with this and other challenges, small water walkers have developed several methods of locomotion at the water interface including: rowing^26–29^, galloping^25,30^, sailing^31^, jumping^23^, meniscus climbing^32^, and Marangoni propulsion^33–35^.

A challenge with fog arises because fog droplets are approximately the same size as the insect’s roughness elements^36^. So, water accumulates in the valleys of the roughness^37^, leading to Wenzel wetting^8^ (no air trapped in the valleys), which results in lower contact angles $\theta _a$ and greater adhesion^7^. This adds weight and increases energy expenditure^38^. As the small fog droplets coalesce, capillary forces can fold thin wing membranes rendering them ineffective for flight^39^. Some fliers, such as mosquitoes, have developed high-acceleration flapping motions that fling droplets from their wings at takeoff to mitigate buildup^40^. Fog can also disturb an insect’s gyroscopic sensors, causing fliers to lose control and fall to the ground^41^.

Rain drops not only pose threats from wetting and surface tension, but their high free-fall velocities and similar size to insect bodies means their inertia is potentially lethal. Yet, small insects like mosquitoes can survive rain-drop collisions in flight due to their strong exoskeleton and low mass^42^. Upon impact with a small insect, the drop loses little momentum and hence imparts a low force. The force increases as insect size increases reaching a maximum of $5\times 10^4$ dyn, the force on an unyielding surface^43^. Even avoiding direct impact, passing droplets generate hazardous disturbances in the air that fliers must mitigate^44,45^.

Given all the threats that insects must deal with to live around water, we find that drain flies are particularly well adapted to handle them. Of the nearly 2900 described species of drain flies worldwide^46,47^, most live in aquatic or semi-aquatic habitats including: in wet woodland patches^48^, near leaking septic lines, in wet shaded areas where mold and algae grow^49^, and in our homes. In this study we report on our investigations into the micro- and nano-structures found on the dense array of hairs that cover drain fly bodies and endow them with superhydrophobicity. These specialized hair structures play a central role in their ability to live in damp conditions and escape from the water hazards that confront them daily.

## Results and discussion

We first examine the drain fly’s morphology. We then theoretically and experimentally study how this morphology decreases wetting in various wetting circumstances. Finally, we study the interaction of live drain flies with water as we confront them with various water threats that approximately simulate real life encounters.

### Body morphology

**Figure 1:**
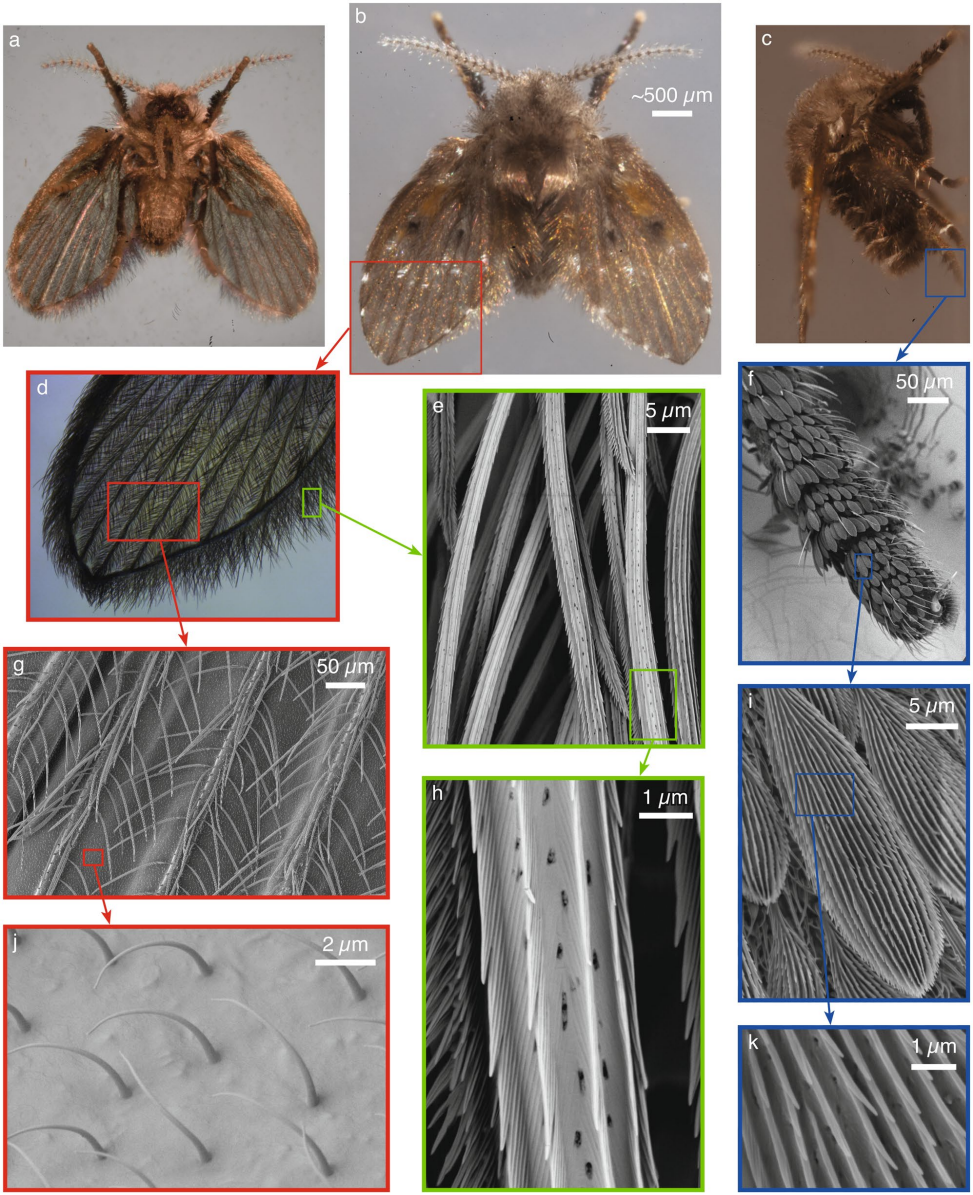
Photos of a drain fly are shown from (**a**) the bottom (ventral, focus stack), (**b**) top (dorsal), and (**c**) side (focus stack). Hair covers the wings (**d**) (focus stack) with the macrotrichia protruding from the veins forming a crisscross pattern (**g**) (multiple macrotrichia are knocked off). Microtrichia lie below the macrotrichia on the wing membrane (**j**). Macrotrichia also densely cover the edge of the wings (**e**) and the fine structures of the macrotrichia are shown in (**h**). Leaflike hairs cover the drain fly’s legs (**f**) and more zoomed in views of these hairs are shown in (**i**) and (**k**).

A drain fly’s body divides into the head region, thorax, and abdomen and is approximately $2.61 \pm 0.36$ mm long and $1.14 \pm 0.19$ mm wide with a mass of $1.9 \pm 0.4$ mg (mean ± standard deviation) (Fig. 1a–c). The head comprises compound eyes, a pair of antennae, and maxillary palps near the mouth (Fig. 1a–c). The thorax bears a single pair of membranous wings (up to 2.9 mm long, 1.5 mm wide, and varying between 0.8 and 8.8 $\upmu \hbox {m}$ thick) that are held flat, covering the thorax and abdomen at rest. Six legs extend from the bottom of the thorax.

Three different types of hair densely cover the fly’s entire body and appendages. The macrotricha are large hairs that predominantly populate the wings, extending to $104 \pm 20\,\upmu \hbox {m}$ in length with a diameter of $2.7 \pm 0.4\,\upmu \hbox {m}$. They protrude upward at an angle from sockets on the veins with approximately $14 \,\upmu \hbox {m}$ spacing, and overlap to form a crisscross pattern that protects the wing membrane as shown in Fig. 1d,g. Along the edge of the wing, the macrotrichia become longer and much denser with approximately $7.9 \pm 1.4\,\upmu \hbox {m}$ spacing (Fig. 1e). The legs also possess sparse scatterings of hairs very similar to the macrotrichia. A second type of large hair is similar to the macrotrichia, but oblong in shape with a flattened, leaf-like appearance ($84.4 \pm 8.9\,\upmu \hbox {m}$ long and $9.2 \pm 1.1\,\upmu \hbox {m}$ wide) (Fig. 1f,i). These hairs are most prominent on the legs (Fig. 1f), the antennae, and in patches along the widest region of the wing. The smallest hairs are the microtrichia, which protrude from the wing membrane, legs, and antenna and lie underneath the larger macrotrichia and leaf-like hairs (Fig. 1j,i). Their conical shape protrudes $4.28 \pm 0.24\,\upmu \hbox {m}$ in length with a diameter of $0.39 \pm 0.02\,\upmu \hbox {m}$ at the base and $0.14 \pm 0.01\,\upmu \hbox {m}$ at the tip (Fig. 1j). Spaced 3–4 $\upmu \hbox {m}$ apart, they bend with random orientations but preferentially toward the distal region of the wing.

As the macrotrichia act as the first and most abundant layer of defense on the wings, which comprise most of the upper surface area of the fly, we investigate their microstructure in more detail. Each hair has approximately ten ridges that start small near the base and grow to a height of approximately 790 nm as they run along the axis. The top of each ridge serrates into conical barbs pointing toward the tip of the macrotrichia (Fig. 1e,h). Shallow nanogrooves decorate the valleys and ridge walls with $98 \pm 10$ nm spacing (Fig. 1h). Small holes ($101 \pm 22$ nm diameter) spaced $780 \pm 183$ nm apart perforate the valleys connecting to the interior of the hollow shaft (dark spots visible in Fig. 1e,h). The leaf-like hairs also possess all the same microstructures, but have many more rows of ridges, due to their larger width (Fig. 1i,k).

The morphology of the hair covering forms an excellent protective layer that helps to keep drain flies dry. In the following three sections we theoretically and experimentally examine the wetting of the wings as an example, but expect similar results on other portions of the body as well due to their similar hair coverings.

### Wetting

#### Millimetric-sized droplets and pools

The drain fly’s hair covering acts as a hierarchical roughness that produces superhydrophobicity. We take the wings as an example and theoretically examine the effect of the drain fly’s hierarchical roughness when it comes in contact with a millimetric-sized droplet or pool of water. The individual macrotrichia act as the peaks of the largest roughness elements, such that the tight spacing allows water to form capillary bridges between hairs. The macrotrichia cover approximately 70% of the projected surface area of the wing, providing a solid-liquid contact fraction of $f_{macro} = 0.7$. The steepness of the valley walls on each macrotrichia also likely enables capillary bridges to form between the ridges. The peaks of these ridges cover approximately 10% of the outer circumference of the hair; $f_{ridge} = 0.1$. Finally, the barbs that serrate the ridges reduce the contact area on the ridges to approximately 25–75% ($f_{barb} = 0.25 - 0.75$), with lower values occurring as the barbs become pointier at the hair tip. Hence, the total solid–liquid contact fraction is $f_s = f_{macro} f_{ridge} f_{barb} = 0.0175 - 0.0525$. The Cassie–Baxter equation^9^ for calculating the apparent contact angle $\theta _a$ is

1 $$\begin{aligned} cos\theta _a = f_s cos\theta _o - f_g, \end{aligned}$$

where $f_g$ is the liquid–gas contact fraction beneath the drop, and $\theta _o$ is the chemical contact angle. Making the simplifying assumption that $f_g = 1 - f_s$ this reduces to

2 $$\begin{aligned} cos\theta _a = f_s(1 + cos\theta _o) - 1. \end{aligned}$$

Using the chemical contact angle of chitin, $\theta _o= 105^{\circ }$, which commonly composes insect cuticles^2,50,51^, the apparent contact angle calculates to $\theta _a = 164^{\circ } - 171^{\circ }$. Further assuming that a droplet only contacts the upper one third of each macrotrichia, reduces $f_s$ to approximately 0.0058 which yields $\theta _a = 175^{\circ }$.

**Figure 2:**
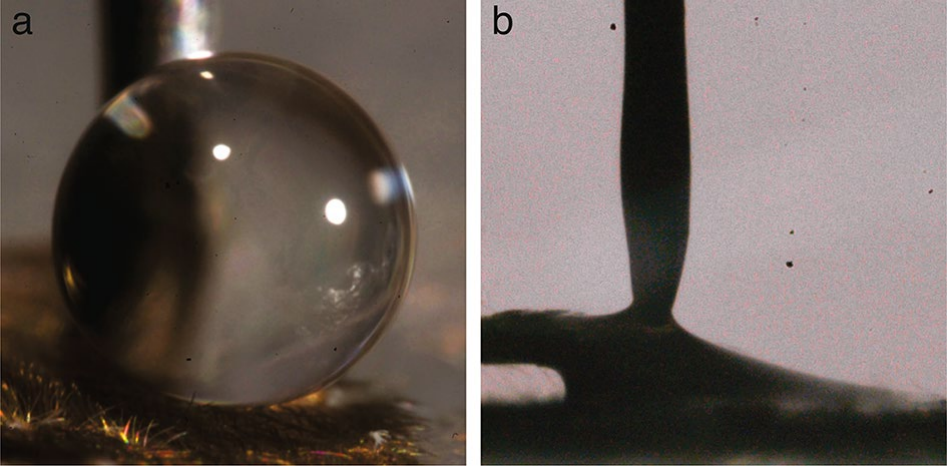
The contact angles of water and silicone oil with a drain fly wing are shown. (**a**) A droplet of water on the wing of a dead drain fly produces a contact angle near $180^{\circ }$. (**b**) When a syringe deposits a droplet of 5 cSt silicone oil, it quickly spreads on the wing and drains over the edges with a contact angle near $0^{\circ }$.

When we place a droplet of water on a fly’s wing, as shown in Fig. 2a, we see that the droplet maintains a spherical shape and that $\theta _a$ lies near 180$^{\circ }$. This shows that a Cassie–Baxter wetting state must exist and that the hierarchical roughness of the drain fly helps it stay dry by minimizing its solid–liquid contact fraction. If the droplet detaches from the syringe, it quickly slides to the side and falls off the wing. We see similar superhydrophobicity on the fly’s body and legs as well, verifying that the covering of leaf-like hairs on the legs follows similar physics resulting in high $\theta _a$.

As drain flies often encounter impure water, we also test their contact angle when a surfactant (sodium dodecyl sulfate, SDS) is present. At low surfactant concentrations ($1.00 \times 10^{-3}$ mol/L and $4.00 \times 10^{-3}$ mol/L) below the critical micelle concentration (CMC, $9.97 \times 10^{-3}$ mol/L^52^), the surface tension of the solutions decreases below the surface tension of water (to $\sigma = 63$ mN/m and 46 mN/m, respectively^52^), but $\theta _a$ is still near 180$^{\circ }$. At 150% of the CMC ($\sigma \approx 39$ mN/m) we see the contact angle decrease to 140$^{\circ }$. Similarly, dish soap, which contains surfactants and other compounds, does not decrease $\theta _a$ at low concentrations (1 drop per 100 mL), but at higher concentrations (6 drop per 100 mL) the wing wets and $\theta _a$ decreases to approximately 45$^{\circ }$. Hence, we see that drain flies’ hair covering enables them to stay dry even in the presence of low concentrations of surfactants.

Drain flies’ liquid repellency appears to be specific to water, with lower surface tension liquids causing wetting. When a droplet of 5 cSt silicone oil ($\sigma = 20$ mN/m) comes in contact with the wing, it quickly penetrates the hairs (no entrapped air, i.e. Wenzel wetting), spreading horizontally through them until it covers the entire wing surface and drains over the edges as shown in Fig. 2b. Similar results occur for olive oil ($\sigma = 36$ mN/m), ethanol ($\sigma = 22$ mN/m), and PP1 (perfluoro-2-methylpentane, $\sigma = 11$ mN/m) showing that although the special morphology of drain flies induces superhydrophobicity it does not induce omniphobicity.

#### Micron-sized droplets

**Figure 3:**
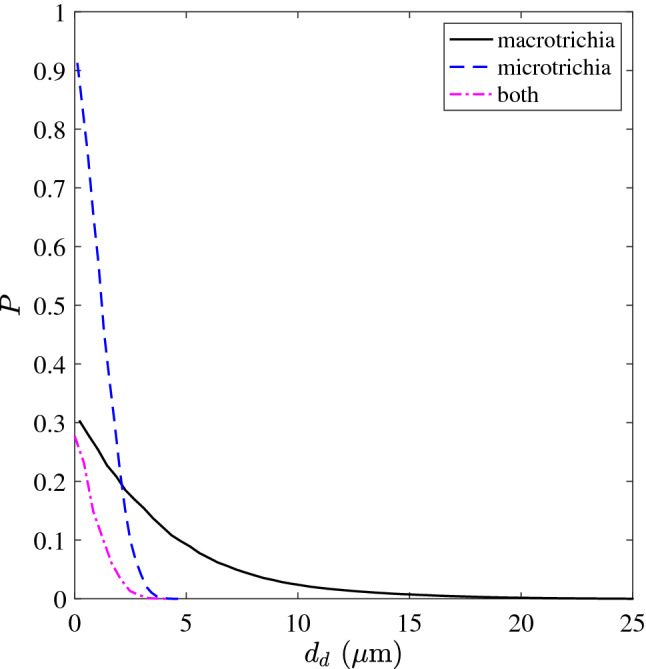
The probability *P* that a droplet of diameter $d_d$ will pass through the wing’s macrotrichia (solid black line), just the microtrichia (dashed blue line), or through both hair layers (dash-dotted magenta line) without contacting any hairs. The black and blue lines are found by convolving various diameter circular disks over binary images of the macrotrichia and microtrichia coverings, similar to Fig. 1d,j, respectively. The magenta line is the product of the other two lines.

As droplets become sufficiently small they can pass between the hairs and reach the main body and appendages. Once again, we take the upper surface of the wings as an example. Figure 3 shows that the small spacing between the macrotrichia (Fig. 1d,g) inhibits droplets with diameters $d_d$ larger than $25 \,\upmu \hbox {m}$ from passing. As the droplet size decreases towards zero the probability of passing the macrotrichia layer increases to 30% $(1 - f_{macro})$ (Fig. 3, solid black line; calculation explained in caption). Having passed the macrotrichium layer, a droplet must also pass the smaller but more tightly packed microtrichia (Fig. 1j) to wet the wing membrane. The microtrichia inhibits all droplets larger than about $4.5 \,\upmu \hbox {m}$ from passing with the probability that smaller droplets can pass and wet the membrane increasing sharply to 91% ($1 - f_{micro}$, where $f_{micro} = 0.09$ is the projected surface area fraction of the microtrichia covering) (Fig. 3, dashed blue line). The combined probability of the water droplets passing both hair layers is shown in Fig. 3 by the dash-dotted magenta line, which indicates that only droplets smaller than $4.5 \,\upmu \hbox {m}$ have any chance of wetting the wing membrane with a maximum chance of 27% ($(1 - f_{macro})(1 - f_{micro})$) for the smallest droplets. Droplets that contact the wing membrane (or other body parts) as opposed to the hairs, wet more surface area (shown in the next section) making liquid removal more difficult.

#### Condensation

**Figure 4:**
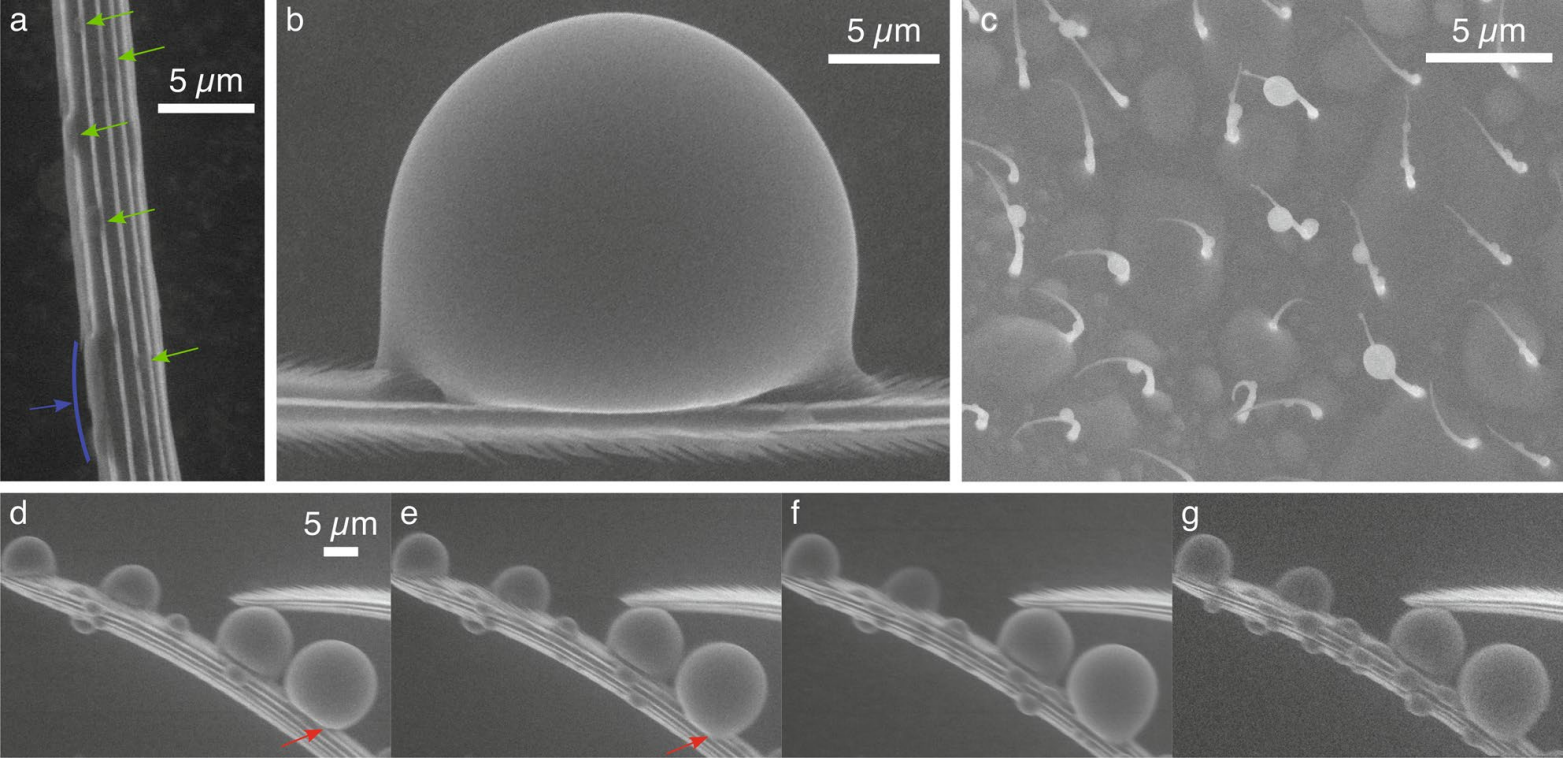
Environmental scanning electron microscope (ESEM) images showing the condensation of droplets on the hairs. (**a**) Condensation initiates in the nano grooves on a macrotrichium (indicated by the green and blue arrows), which grow into spherical droplets (**b**). (**c**) Condensation also occurs on the wing membrane and microtrichia, where more spherical droplets form. In (**d**)–(**g**) a sequence is shown of droplets growing over time (4–6 s between frames) as condensation continues. The red arrows indicate a droplet stepping between ridges.

One final way for water to collect on a drain fly is by condensation at the dew point. Figure 4 shows the process of condensation of water on various wing surfaces inside an environmental scanning electron microscope. On the macrotrichia (Fig. 1h), condensation initiates in the valleys (Fig. 4a), likely due to the lower energy requirement in nanoscale V-shaped structures^53^. In the early stages, water collects as spherical sections and elongated filaments as indicated in Fig. 4a by the green and blue arrows respectively and previously studied for synthetic structures^54^. As water continues to condense, the volume increases so much that a groove cannot confine a growing droplet which bulges out forming a more spherical shape (Fig. 4b). The nanogrooves inside the valleys (Fig. 1h) and the barbs on the ridges pin the contact line in both the axial and circumferential directions allowing droplets to exhibit a range of local contact angles as seen on the left and right of the droplet in Fig. 4b. Further growth causes the droplets to step from one sharp edge to another as indicated by the red arrows in the image sequence shown in Fig. 4d–g. Water also collects as small droplets on the microtrichia and as droplets that grow and combine to form pools on the wing membrane (Fig. 4c). The collection of water on the macrotrichia and microtrichia results in more spherical droplets than collection on the wing membrane. This decreases water’s contact area allowing for easier removal upon experiencing an acceleration.

### Water threats and encounters with live drain flies

To observe the wetting properties of drain flies in practice, we investigate the fluid dynamics as water comes in contact with live drain flies and comment on the flies’ typical reaction to various threats, as summarized in Table 1. We first look at millimetric-sized droplets, approximating the fly’s interaction with rain, dripping liquid, and sprays from faucets or shower heads. Next, we study mists to approximate natural fog and steamy bathrooms. Finally, we examine the fly’s interaction with pools and small waves to simulate ponds, puddles, streams, toilets and water in the P-trap of drains.

**Table 1:** Summary of drain fly behavioral response when confronted with threats, showing the number of trials for each threat, the number of observations of the dominant response, the percent occurrence of that response, and the number of flies tested for that threat. $^{\text {a}}$The one case in which the fly begins to flee before impact of a single large droplet is the event shown in Fig. 5a.

Threat event	Behavioral response	No. trials	No. responses	%	Flies tested
Single large droplet impact (Fig. 5b)	Flees after impact	8	7^a^	88	5
Small droplets impact (Fig. 5c)	Flees after impact	14	12	86	3
Multiple droplets impact (Fig. 5d)	Recovers after impact	7	7	100	2
Pinned by non-water droplet (Fig. 5e)	Tries to escape	6	6	100	4
Surrounded by mist (Fig. 6a)	Takes flight after $\sim 3$ min	15	15	100	3
On pool surface (Fig. 7a)	Takes flight in $\lesssim 1$ s	8	8	100	2
Submerged in pool undisturbed	Minimal attempt to escape	13	13	100	10

#### Droplets

**Figure 5:**
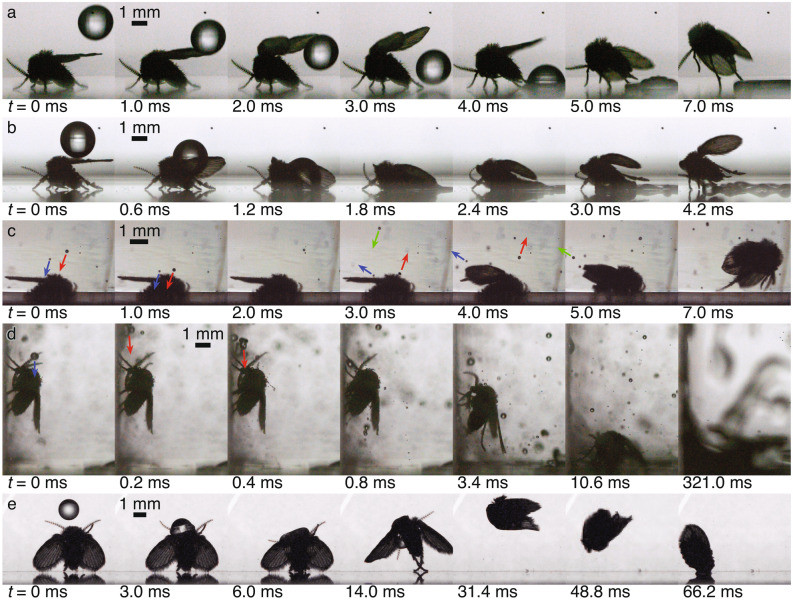
Various encounters between millimetric-sized droplets and drain flies are shown. (**a**) A single 2.2 mm droplet impacts at 0.95 m/s directly behind a fly standing on the floor causing it to flee. (**b**) A 2.2 mm droplet impacts on top of a fly at 1.89 m/s smashing it onto the ground. (**c**) Three small droplets with diameters between 0.13 and 0.20 mm impact and rebound on a fly’s wings at 1.87–2.91 m/s causing it to flee (trajectories are indicated by the arrows). (**d**) A 0.5 mm droplet (trajectory shown by the blue arrow) impacts the fly’s antenna at 7.70 m/s followed by a cluster of three similar sized droplets (trajectory shown by red arrows) that knock the fly off the wall. After several more impacts, the fly (out of focus) pulls itself out of the puddle and walks away. (**e**) A 1.7 mm droplet of 5 cSt silicone oil impacts a fly’s head at 0.48 m/s causing it to jump and land on its head, adhering itself to the ground, where it died. Supplementary videos 1–5 show panels a–e, respectively.

When droplets of water fall towards a fly, we observe different droplet-fly interactions depending on the droplet size, velocity, and quantity. We first examine a single droplet falling towards a fly from above. As the fly in Fig. 5a sits on the floor, a 2.2 mm diameter droplet falls toward it at 0.95 m/s. The fly senses its approach, either visually^55^ or with sensilla^56^ that feel the air flow, and begins to raise its wings preparing for takeoff ($t = 1$ ms). The droplet impacts the floor behind the fly as it leaps into the air for a successful escape. In Fig. 5b, a similar set of circumstances occurs except this time the droplet falls from directly above the fly with twice the velocity. The fly does not respond to the threat before impact and the droplet smashes the fly into the ground spreading over the upper surfaces of the fly ($t = 0.6$–1.8 ms). The fly’s superhydrophobic hair covering causes the droplet to quickly glide off the wing onto the floor and the fly leaps into flight, which is the typical response (Table 1), with the only visible damage being the loss of some hair ($t = 2.4$–4.2 ms).

A spray of droplets can be more or less harmful to a fly than a single droplet because of the various droplet sizes and velocities present. In Fig. 5c, a spray of numerous droplets approaches a fly standing on the floor. Three small droplets (0.13–0.20 mm diameters) with relatively low velocities (1.87–2.91 m/s) lead the group and impact first, rebounding off the fly ($t = 0$–5.0 ms). These impacts do not harm the fly but alert it to potential danger and induce it to flee ($t = 4.0$–7.0 ms). Table 1 shows that although it is possible for the flies to sense incoming threats (Fig. 5a) they typically do not react until after droplets impact them (Fig. 5b,c). Once droplet impact occurs the flies react quickly. We measure their reaction time as the time from impact to the moment they begin to raise their wings to flee to be $4.4 \pm 1.3$ ms (mean ± standard deviation, from 10 trials).

In another case, we observe that sometimes the flies’ reaction time is not fast enough to avoid additional impacts. In Fig. 5d, a spray of droplets approaches a fly standing on the wall. This time the first impact occurs on the fly’s antenna with a 0.5 mm droplet at 7.70 m/s ($t = 0$–0.2 ms, blue arrow). A cluster of three similar sized droplets quickly follows ($t = 0.2$–0.4 ms, red arrows), impacting the fly on the head, splashing ($t = 0.8$ ms) and knocking it off the wall ($t = 3.4$–10.6 ms). The fly endures multiple additional impacts (Supplementary video 4). When the spray terminates, the fly gradually works its way out of the newly formed puddle ($t = 321$ ms) and walks away without the appearance of major damage, but with a much slower gait than normal. We find that flies can typically recover from this type of attack (Table 1).

From the multiple videos of water droplet impacts taken, we see that the drain flies always stay dry (i.e., water does not adhere to them) and can flee and recover from the first few impacts. After several repeated droplet impacts, the flies begin to move slower and often incur damage to their thin appendages, such as torn wings, damaged legs, or broken antennae. In one case we subjected a fly to repeated large droplet impacts (about six), similar to Fig. 5b, until it became unresponsive to additional impacts and eventually died. We expect the threshold for the number of impacts to cause injury or death to vary with droplet size, velocity, and impact location and orientation.

If the fly comes in contact with a droplet of another liquid, the results differ greatly. Figure 5e shows a 1.7 mm droplet of 5 cSt silicone oil impacting a fly at a low velocity of 0.48 m/s. As the fly stands against the tank wall ($t = 0$ ms), the silicone oil impacts its head ($t = 3.0$ ms), wetting the fly ($t = 6.0$ ms). The fly jumps, turns in the air, and lands on its head ($t = 14.0$–66.2 ms). The droplet adheres the fly to the floor and the fly jerks repeatedly trying to escape without success (Supplementary video 5). The fly died. Similar experiments were performed with olive oil, ethanol, and PP1 with the same results for each (Table 1) except that the fly did not die when impacted with PP1. This likely occurs because the high volatility of PP1 causes the droplet to evaporate quickly, freeing the fly.

#### Mist

**Figure 6:**
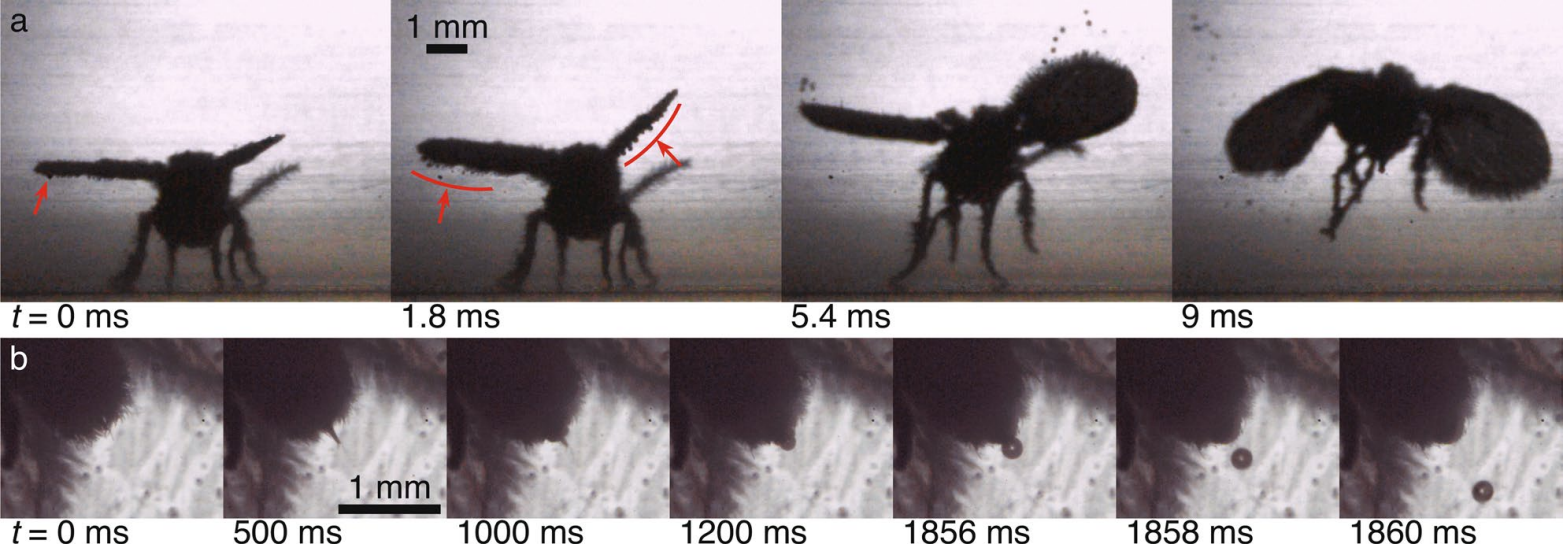
Drain fly behavior in mist is shown. (**a**) A drain fly that has been sitting in mist for several minutes has droplets collected on its hair (indicated by red arrow), which fling away as it flaps its wings. (**b**) A fly sitting in fog urinates, ejecting a single 0.2 mm diameter droplet at 0.18 m/s. Supplementary videos 6–7 show panels a–b respectively.

When a drain fly sits in mist, water gradually collects on its hair (through droplet impacts and condensation) and droplets smaller than $4.5 \,\upmu \hbox {m}$ have a small chance of passing through the hair covering to wet the wing membrane (discussed in the “Micron-sized droplets” section above). Figure 6a shows a drain fly that has been sitting in mist with a mean droplet diameter of approximately $10 \,\upmu \hbox {m}$ for several minutes. Hence, there should be minimal wetting to the wing membrane from micro droplets. This mist approximates natural fog which has a mean droplet diameter between 8 to $24 \,\upmu \hbox {m}$^36^. Small droplets collect on the macrotrichia at the edge of the wings (Fig. 6a, $t=0$ ms). As is typical of our observations, the mist eventually induces the fly to move and it takes flight (Table 1). The flapping of its wings generates accelerations, *a*, up to 400 *g* at the wing tips. A simple force balance, $m_d a = \sigma L$ (where $m_d$ is the mass of a spherical droplet, $\sigma$ is the surface tension coefficient, and *L* is the droplet-hair contact length, which we approximate as the macrotrichium diameter) shows that such high accelerations should remove droplets larger than approximately $45 \,\upmu \hbox {m}$. Smaller contact lengths would allow the removal of even smaller droplets, further drying the fly and minimizing its effective mass even more. The collection of mist droplets on the hair and the tendency to occasionally take flight help to keep the drain fly dry. The negative effects of wing folding and to flight control that mosquitoes experience^39,41^ are not seen in drain flies.

Mist may also aid drain flies in water intake as it is the only circumstance in which we frequently observed drain flies urinating (caught on video four times, and observed several more). The flies manage to stay dry during urination due to a conical spike and bulge that protrude from the anus. After extending beyond the hair covering as shown in Fig. 6b ($t = 0$–1000 ms), the fly excretes a 0.2 mm droplet while it retracts the spike ($t = 1200$–1856 ms). The droplet launches away from the fly, with sufficient velocity to avoid further contact and self-wetting ($t = 1858$–1860 ms). As the bulge retracts, the spike reemerges followed by the full retraction of both (Supplementary video 7).

#### Pools and waves

**Figure 7:**
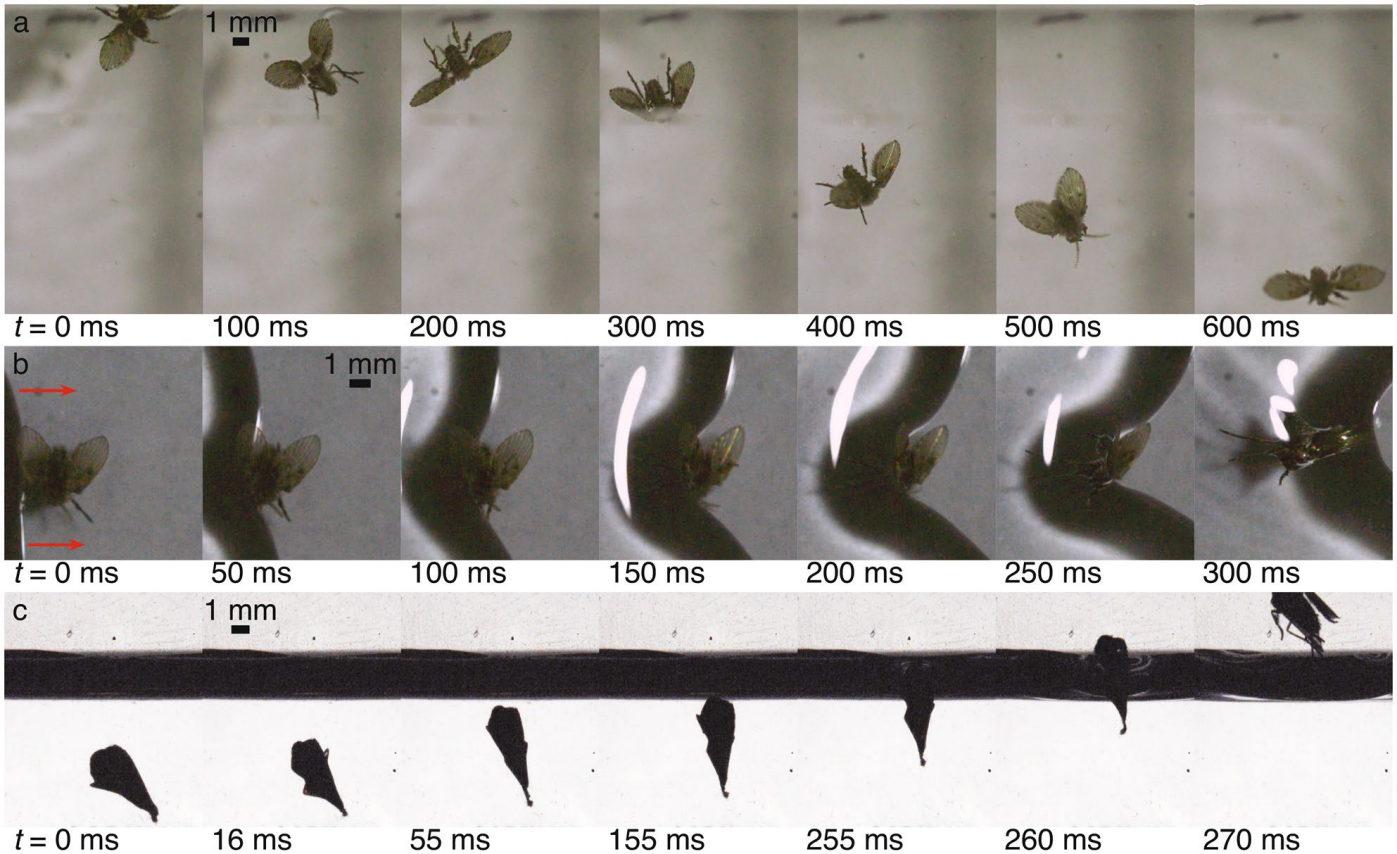
Drain fly interactions with pool surfaces and waves is shown. (**a**) A fly, seen from above, crashes into the container wall at the top of the frame and lands back down on the water surface (just before $t = 0$ ms). The fly rolls ($t = 0$–400 ms), stands on the water ($t = 500$ ms), and jumps to fly away ($t = 600$ ms). (**b**) An approximately 3-mm-high wave traveling left to right (as indicated by the arrows) impacts a fly standing on the floor, passes over the fly and forms a plastron indicated by the shiny appearance of the submerged fly. (**c**) A fly, pinned to the tank wall by its plastron, detaches itself, rises to the surface and escapes. Supplementary videos 8–10 show panels a–c, respectively.

Drain flies’ erratic manner of flight can lead to collisions that knock them out of the air and onto the surface of a pool. Figure 7a shows the typical situation following such an impact. As the fly drifts away from the collision site, it rolls on the pool making several attempts to stand ($t = 0$–400 ms). The fly manages to stand in less than a second and quickly leaps into flight ($t = 500$–600 ms) by moving its four hind legs in a rowing-like jump, similar to that of a water strider^11,28^ (Supplementary video 8; the jumping motion is smoother in some other observations). In all observations, the flies never stay on the pool surface for more than a couple of seconds (Table 1) and do not exhibit any kind of water walking motion, seeming to prefer to locomote in the air and rest on solid surfaces.

A drain fly’s ability to support its weight on and leap from the pool surface comes from its small size. The Baudoin number is the ratio between the force of gravity and the maximum surface tension (when the force vector points directly up) and is defined as $Ba=m_f g/\sigma P$, where $m_f$ is the fly’s mass, *g* is the acceleration of gravity, $\sigma$ is the surface tension coefficient, and *P* is the perimeter of the depression in the pool surface. With $m_f = 1.9$ mg and $P = 7.7$ mm for the contacting portions of all six legs, $Ba = 0.034$ for a fly in the standing position. This means that surface tension can exert a force up to 30 times (1/*Ba*) the weight of the fly explaining their ability to stand on the surface. The perimeter *P* increases for other fly orientations on the surface, increasing the safety margin (e.g., on its wings, back, and antennae $P = 22.2$ mm and $1/Ba = 86$).

As a drain fly stands near a lapping pool of water, oncoming waves can threaten to maul or submerge them, if they do not fly away to safety. In Fig. 7b the fly does not move as the wave impacts its right legs, pins them to the floor, and passes over the top ($t = 0$–100 ms). The wave continues over the body and wings, only contacting the tops of the hairs, and entrapping a thin air layer, known as a plastron, which appears like a bubble surrounding the fly ($t = 150$–300 ms). The plastron allows the fly to breath while submerged^57,58^, but also pins the fly to the floor or wall of the container, thwarting its escape.

If the drain flies remain undisturbed in water, they do not appear to attempt to escape and eventually die (Table 1). For an insect to survive submerged indefinitely, the surface area of its plastron must be large enough that the rate of $\hbox {O}_2$ and $\hbox {CO}_2$ exchange with the water suffices to meet the insect’s metabolic needs^59^. We found that the survival rate for the drain flies decreases with submergence time. All drain flies submerged for less than 5 h lived; 3 survived 3 h of submergence, and 4 others survived shorter test durations (100% survival). Following approximately 5 h of submergence, 2 of 4 flies died and 2 lived at least 24 h after release (50% survival rate). Two flies left submerged overnight died. We observed that the drain flies occassionally move their appendages and deform the plastron walls while submerged. Previous researchers^60,61^ explain that this behavior can increase fluid flow over the plastron, and hence $\hbox {O}_2$ and $\hbox {CO}_2$ exchange, helping insects survive for a longer submergence duration.

Although submergence by a wave can kill drain flies, it does not necessarily constitute a death sentence. In Fig. 7c we see another fly encapsulated in a plastron, submerged in a pool, and pinned to the container wall. To see if the flies have the ability to escape, in this case we stimulate action by tapping lightly on the container encouraging the fly to move. The fly gradually travels diagonally upwards over a distance of approximately 10–20 mm until it arrives in the position seen at $t = 0$ ms. One final exertion by the fly at $t = 16$ ms dislodges it from the wall allowing it to rise to the surface ($t = 55$–155 ms). Upon contacting the surface, the plastron pops, surface tension launches the fly upwards into the air ($t = 255$–270 ms), and the fly emerges unharmed.

From this event we see that a drain fly with a plastron is buoyant, and simple calculations show that the ratio of the buoyant force to the fly’s weight is $F_b/m_f g \approx 1.5$–2.7 (assuming axisymmetry and an equilateral triangle cross section for volume calculations of the submerged fly; e.g., Fig. 7c, $t = 55$ ms). A drain fly’s plastron gradually dissolves and when fully dissolved, the fly sinks. This shows that the plastron not only allows the fly to breath while submerged, but enables it to rise to the pool’s surface as long as it does not pin the fly to a solid surface.

## Conclusions

We have studied how drain flies survive in wet conditions and manage many water related threats. Like many other insects, superhydrophobicity is key to drain flies’ survival in their preferred aquatic or semi-aquatic habitats. If their hydrophobicity were to be removed, they would easily wet, water’s surface tension would hold them fast, and they would die, as demonstrated herein with other wetting liquids. Drain flies’ superhydrophobicity comes from a combination of their sufficiently high chemical contact angle and the three-fold hierarchical roughness found in their unique hair covering that produces an apparent contact angle near $180^{\circ }$. The roughness of the hair covering ranges in scale from the micron-sized arrays of the macrotricha and leaf-like hairs, down to the nanoscale ridges, valleys, barbs, and grooves found on each hair. The microtrichia provide a secondary layer of defense that helps the flies stay dry when they lose some of the larger hairs or are confronted with condensation or the micron-sized droplets found in mist.

Live drain flies prove to be very resilient to the various water threats they encounter. Droplets impacting drain flies exhibit rapid lateral spreading, retraction, and even rebound, similar to impact on other superhydrophobic surfaces. If large impacts prove unavoidable the drain flies may sustain minor injuries to their thin appendages and even die. Mist gradually accumulates into larger droplets on the flies, but the hair covering traps the majority of the water. It adheres to the hairs in a spherical shape that more easily detaches when subjected to sufficient accelerations, such as flapping of the drain fly’s wings. When needed, drain flies can also stand on and leap from the surface of a pool of water. If they become submerged, their hair covering entraps a thin air layer (a plastron) over the surface of their entire body. This air layer decreases their effective density enabling them to float to the surface of the pool, where they can escape. If they become pinned to a solid beneath the water surface, the air layer combined with the exchange of $\hbox {CO}_2$ and $\hbox {O}_2$ with the water, enables the flies to survive submerged for up to approximately 5 h.

We see that drain flies’ specialized hair covering combined with their rapid flight from threats enables them to stay dry and safe in both the wet environments of our homes and in nature. This new understanding of the unique morphology of drain flies’ hair structure may aid in the design of future superhydrophobic surfaces or the development of appropriate wetting pesticides.

## Materials and methods

### Insect collection and sample preparation

Adult drain flies were collected from houses (bathroom, kitchens or near other damp locations) at KAUST using narrow mouth plastic bottles. They were transported to the laboratory and maintained at $20^{\circ }\hbox {C}$. Live insects were used for water threat and encounter studies within 48 h of capture. To collect wing samples for imaging purposes, specimens were sedated using formaldehyde fumes. The samples were then dried at room temperature inside a laminar flow cabinet and stored in paraffin sealed petri plates for later use. Wings were then carefully dissected using a surgical scalpel and reattached to glass slides using double-sided tape. To remove hair from the wing surface, the wing was sprayed with ample amounts of Milli-Q water.

### Photography and optical microscopy

Wings flattened on microscope slides were imaged with a Zeiss Axioscope microscope with a Zeiss AxioCam ERc 5s camera attached. For higher magnification images of the body surface and hair, we used a Nikon SMZ25 Stereomicroscope. Images were also taken with a Nikon D3X SLR camera and Leica Z16 APO lens. Wing dimensions and other body measurements were estimated using the built-in scale, ImageJ, or MATLAB with the mean and standard deviation of several measurements reported herein. High speed photography was accomplished with a color Phantom V710 camera. Focus stacking for the images in Fig. 1a,c,d was accomplished in Adobe Photoshop. The histograms of many of the SLR and high-speed photography images were adjusted in Adobe Lightroom to correct the white balance and provide minor brightening.

### Scanning electron microscopy and environmental scanning electron microscopy

Body surfaces and hair structures of the drain fly were examined with scanning electron microscopy (SEM). For the analysis, body parts (head, antennae, legs and wing) were dissected carefully from dead individuals and mounted on aluminum stubs using a carbon tape and then sputter-coated with a 4 nm Au layer. The images were obtained on Quanta 600 microscopes. Measurements were made from digital images using the ImageJ software.

The detailed analysis of the wetting phenomena and behaviors of condensed water droplets on the wing surface were observed using Quanta 600 SEM (Thermo Fisher Scientific) equipped with Environmental SEM (ESEM) mode including built-in cooling stage and gaseous secondary electron detector. The wing samples were attached to aluminum stubs using double-sided copper tape. We initiated water droplet formation on the wing surface by gradually increasing the water vapor pressure inside the SEM chamber from 600 Pa to 820 Pa at $2^{\circ }\hbox {C}$ stage temperature. Images were captured every 2 s intervals at an accelerating voltage of 7 kV.

### Measurement of the contact angle

The advancing apparent contact angle $\theta _a$ of several liquids was measured on drain flies’ wings using the sessile drop method. Water ($\sigma = 72$ mN/m), 5 cSt silicone oil (from Clearco Products, $\sigma = 20$ mN/m), olive oil ($\sigma = 36$ mN/m), ethanol ($\sigma = 22$ mN/m), and PP1 (perfluoro-2-methylpentane, $\hbox {C}_6\hbox {F}_{14}$, from FLUTEX, $\sigma = 11$ mN/m), where used. Contact angles of solutions of water with SDS (sodium dodecyl sulfate) and water with Pril dish soap (by Henkel, contains anionic and amphoteric surfactants), were also measured. The measurement was performed as follows. A droplet of liquid was formed just above the drain fly and expanded until it came in contact with the fly. Images of the contact angle with water were taken with a Nikon D3X SLR camera with a Leica Z16 APO microscope lens. Images of the contact angle for 5 cSt silicone oil, olive oil, PP1, ethanol, and water-surfactant mixtures were taken with a color Phantom V710 high-speed camera.

### Simulating water encounters and threats

To investigate the dynamic wetting behavior of the liquid and the flies’ response to various water threats, we contained each fly in a glass or acrylic tank ranging in size from $3.3 \times 3.5 \times 3.5\;\hbox { cm}^3$ to $11.6 \times 11.6 \times 21.6\;\hbox { cm}^3$ to a Petri dish of 8.7 cm diameter and 1.5 cm height. After giving the fly sufficient time to acclimate to the surroundings we confronted the fly with one of various water threats and filmed the encounter with a color Phantom V710 high-speed camera at up to 5000 frames per second. From these videos, we observed the flies’ response, wetting properties, and measured droplet diameters and velocities. Multiple drain flies were used for the various tests and several repetitions of each of the tests were performed as summarized in Table 1.

#### Experiments on fly interactions with droplets and mist

We studied how the flies interact with droplets in three different ways. First, we introduced single-droplet threats by inserting a syringe of liquid inside the tank above the fly and squeezing out a droplet that landed on or near the fly. Second, we shot a spray of droplets at the fly using a household spray bottle that formed a range of droplet diameters and velocities. Finally, to generate fog we used a Proton Ultrasonic household humidifier to make micrometer-sized droplets that flowed into the tank through tubing, filling the entire volume of the tank with a cloud.

#### Experiments on fly interactions with pools and waves

We also investigated how drain flies interact with a pool in three different ways. First, we filled the bottom of the tank with water. As the flies often land on the sides of the tank and sit for long periods of time, we lightly tapped the tank walls to induce them to fly. Due to their erratic flying behavior they would often land, fall, or crash into the pool surface. Second, after placing a fly in a Petri dish, we inclined it and filled the lower section with water. When the fly landed on the dry ground, we laid the dish down flat, forming a wave directed at the fly. Finally, after submerging a fly in the pool we observed its behavior.

## Supplementary information

Supplementary Video 1.

Supplementary Video 2.

Supplementary Video 3.

Supplementary Video 4.

Supplementary Video 5.

Supplementary Video 6.

Supplementary Video 7.

Supplementary Video 8.

Supplementary Video 9.

Supplementary Video 10.

Supplementary Legends.
