# Author Correction: How drain flies manage to almost never get washed away

**DOI:** 10.1038/s41598-021-86046-z

**Published:** 2021-03-23

**Authors:** Nathan B. Speirs, Gauri A. Mahadik, Sigurdur T. Thoroddsen

**Affiliations:** Division of Physical Science and Engineering, King Abdullah University of Science and Technology (KAUST), Thuwal, 23955‑6900 Saudi Arabia

Correction to: *Scientific Reports* https://doi.org/10.1038/s41598-020-73583-2, published online 20 October 2020

The original version of this Article contained errors, where the identification of the drain fly individuals was incorrect. In the Abstract,

“Drain flies, *Pshycoda* spp. (Order Diptera, Family Psychodidae), commonly reside in our homes, annoying us in our bathrooms, kitchens, and laundry rooms.”

now reads:

“Drain flies, Psychodidae spp. (Order Diptera, Family Psychodidae), commonly reside in our homes, annoying us in our bathrooms, kitchens, and laundry rooms.”

Additionally, in the Materials and Methods, under the subheading ‘Insect collection and sample preparation’,

“Adult drain flies (*Psychoda* spp.) were collected from houses (bathroom, kitchens or near other damp locations) at KAUST using narrow mouth plastic bottles.”

now reads:

“Adult drain flies were collected from houses (bathroom, kitchens or near other damp locations) at KAUST using narrow mouth plastic bottles.”

These errors have now been corrected in the PDF and HTML versions of the Article.

