# Supplementary material for: How drain flies manage to almost never get washed away
Source: Sci Rep. 2020 Oct 20;10:17829. doi: 10.1038/s41598-020-73583-2 (PMC7575561; doi:10.1038/s41598-020-73583-2)
Supplement: Supplementary file 11 — Supplementary Legends. [file 41598_2020_73583_MOESM11_ESM.pdf]

How drain flies manage to almost never get washed away

Nathan B. Speirs, Gauri A. Mahadik, and Sigurdur T. Thoroddsen

Supplemental video captions

Supplemental video 1
A single 2.2 mm droplet impacts at 0.95 m/s directly behind a fly that stands on the floor causing it to flee. The video is played back at 0.6% of real speed. Corresponds to Fig. 5a.

Supplemental video 2
A 2.2 mm droplet impacts on top of a fly at 1.89 m/s smashing it onto the ground. The fly then stands and flees. The video is played back at 0.6% of real speed. Corresponds to Fig. 5b.

Supplemental video 3
A fly stands on the floor with a spray of droplets impacting all around. Three small droplets with diameters between 0.13 and 0.20 mm impact and rebound on the fly's wings at 1.87 to 2.91 m/s warning it of danger, without injuring it, and causing it to flee. The video is played back at 0.6 % of real speed. Corresponds to Fig. 5c.

Supplemental video 4
A 0.5 mm droplet impacts the fly's antenna at 7.70 m/s quickly followed by a cluster of three similar sized droplets that knock the fly off the wall. After several more impacts, the fly, which has been pushed out of the focal plane, pulls itself out of the puddle and walks away. The video is played back at 1.2% of real speed. Corresponds to Fig. 5d.

Supplemental video 5
A 1.7 mm droplet of 5 cSt silicone oil impacts a fly's head at 0.48 m/s causing it to jump and land on its head, adhering itself to the ground. After trying to escape without success the fly died. The video is played back at 0.6% of real speed. Corresponds to Fig. 5e.

Supplemental video 6
A drain fly that has been sitting in mist for several minutes has droplets collected on its hair, which fling away as the it flies away. The video is played back at 0.6% of real speed. Corresponds to Fig. 6a.

Supplemental video 7
Flies that sit in fog were frequently found urinating. This fly that had been sitting in fog for several minutes urinates, ejecting a single 0.2 mm diameter droplet at 0.18 m/s. The video is played back at 18% of real speed. Corresponds to Fig. 6b.

Supplemental video 8
A fly, seen from above, crashes into the container wall at the top of the frame and lands back down on the surface of a pool of water. The fly rolls, stands on the water, and jumps to fly away to land on a solid surface, which they prefer. The video is played back at 4.8% of real speed. Corresponds to Fig. 7a.

Supplemental video 9
An approximately 3-mm-high wave traveling left to right impacts a fly standing on the floor, passes over the fly and forms a plastron indicated by the shiny appearance of the submerged fly. The video is played back at 2.4% of real speed. Corresponds to Fig. 7b.

Supplemental video 10

A fly, pinned to the tank wall by its plastron, detaches itself with an aggressive move that deforms the plastron. This allows the fly to rise to the surface and escape. The video is played back at 6% of real speed. Corresponds to Fig. 7c.
